# Peptide bonds affect the formation of haloacetamides, an emerging class of N-DBPs in drinking water: free amino acids versus oligopeptides

**DOI:** 10.1038/srep14412

**Published:** 2015-09-23

**Authors:** Wenhai Chu, Xin Li, Naiyun Gao, Yang Deng, Daqiang Yin, Dongmei Li, Tengfei Chu

**Affiliations:** 1 State Key Laboratory of Pollution Control and Resources Reuse, College of Environmental Science and Engineering, Tongji University, Shanghai, 200092, China; 2 Department of Earth and Environmental Studies, Montclair State University, Montclair, NJ 07043, USA

## Abstract

Haloacetamides (HAcAms), an emerging class of nitrogenous disinfection by-products (N-DBPs) of health concern, have been frequently identified in drinking waters. It has long been appreciated that free amino acids (AAs), accounting for a small fraction of the dissolved organic nitrogen (DON) pool, can form dichloroacetamide (DCAcAm) during chlorination. However, the information regarding the impacts of combined AAs, which contribute to the greatest identifiable DON portion in natural waters, is limited. In this study, we compared the formation of HAcAms from free AAs (tyrosine [Tyr] and alanine [Ala]) and combined AAs (Tyr-Ala, Ala-Tyr, Tyr-Tyr-Tyr, Ala-Ala-Ala), and found that HAcAm formation from the chlorination of AAs in combined forms (oligopeptides) significantly exhibited a different pattern with HAcAm formation from free AAs. Due to the presence of peptide bonds in tripeptides, Tyr-Tyr-Tyr and Ala-Ala-Ala produced trichloroacetamide (TCAcAm) in which free AAs was unable to form TCAcAm during chlorination. Moreover, peptide bond in tripeptides formed more tri-HAcAms than di-HAcAms in the presence of bromide. Therefore, the peptide bond may be an important indicator to predict the formation of specific N-DBPs in chlorination. The increased use of algal- and wastewater-impacted water as drinking water sources will increase health concerns over exposure to HAcAms in drinking water.

As a result of rapid population growth and rising water demand, drinking water source waters are facing threats of insufficiently treated wastewater effluents or algal blooms. These pollution sources are characterized by higher levels of dissolved organic nitrogen (DON) that can potentially react with certain disinfectants (e.g., chlorine) to form unwanted nitrogenous disinfection by-products (N-DBPs) in drinking water treatment plants (DWTPs)^1^,^2^,^3^. Recently, interest in the formation of N-DBPs has increased because toxicological studies have demonstrated that N-DBPs are typically more genotoxic, cytotoxic, or carcinogenic than most carbonaceous disinfection by-products (C-DBPs) that have long been a major focus in previous studies^1^,^4^,^5^. Haloacetamides (HAcAms), an emerging class of halogenated N-DBPs, are of particular concern because they were reported to be very cytotoxic and genotoxic in mammalian cell assays (for example, over 100 times more cytotoxic and 10 times more genotoxic than HAAs)^6^ and were frequently detected in drinking water^2^,^7^,^8^.

The formation of N-DBPs from amino acids (AAs) upon chlorination is of interest, as AAs account for a significant fraction of DON in natural waters. In prior studies, free AAs were mostly selected as model compounds to investigate the DBP formation mechanism^1^,^9^. However, free AAs make up only an insignificant fraction (<6%) of the DON pool; in contrast, combined AAs contribute to the greatest identifiable portion, especially in algal- and wastewater-impacted water^10^,^11^,^12^. Therefore, it is essential to examine the formation of N-DBPs from combined AAs. Combined amino acids (e.g., oligopeptides and proteins) are ubiquitous in surface waters and typically derive from viral lysis or autolysis of bacteria, microbial secretion of extracellular enzymes, atmospheric deposition, or anthropogenic inputs as pollutants^11^,^13^,^14^.

It has been appreciated that part of the free AAs may serve as HAcAm precursors^1^,^15^,^16^. For example, free tyrosine (Tyr) could react with chlorine to form dichloroacetamide (DCAcAm) and trichloroacetamide (TCAcAm)^17^. However, alanine (Ala) cannot form any HAcAm but might serve as a chloroform precursor^15^,^18^. Unfortunately, it was still unclear whether the formation of HAcAms from the chlorination of oligopeptides and free AAs behaves significantly differently due to the presence of peptide bonds in the oligopeptides. The objective of this study was to compare the formation of HAcAms between the chlorination of free AAs and low-molecular mass combined AAs (oligopeptides), and thus evaluate the impacts of peptide bonds on HAcAm formation. Two free AAs, Tyr (HAcAm precursor) and Ala (Non-HAcAm precursor), and four oligopeptides, Tyr-Ala, Ala-Tyr, Tyr-Tyr-Tyr, and Ala-Ala-Ala (Fig. 1) were selected as precursor compounds in this study, because they share similar molecular structures except the presence or absence of peptide bonds on HAcAm formation.

## Methods

### Materials

Chloroacetamide (CAcAm) (98.5%), DCAcAm (98.5%), and TCAcAm (99%) standards were obtained from Alfa Aesar (Karlsruhe, Germany). Bromochloro- (BCAcAm), dibromo- (DBAcAm), bromodichloro- (BDCAcAm), dibromochloro- (DBCAcAm), and tribromoacetamide (TBAcAm) standards were all purchased from Orchid Cellmark (New Westminster, BC, Canada). Bromoacetamide (BAcAm), two haloacetonitriles (HANs) (dichloroacetonitrile [DCAN] and trichloroacetonitrile [TCAN]), and the model compounds (Tyr [≥99%], Ala [≥99%], Tyr-Ala [>98%], Ala-Tyr [>98%], Ala-Ala [>98%], Tyr-Tyr-Tyr [>98%], and Ala-Ala-Ala [>98%]) were purchased from Sigma–Aldrich (Oakville, ON, Canada). A sodium hypochlorite solution (reagent grade [>98%], active chlorine >5%, Sinopharm Chemical Reagent Co., Ltd., China) was used to prepare free chlorine stock solutions. The ultrapure water was produced with a Millipore Milli-Q Gradient water purification system (Billerica, MA, USA). All the other chemical reagents were at least of analytical grade, and obtained from Sinopharm Chemical Reagent Co., Ltd (Shanghai, China) unless otherwise noted.

### Experimental procedure

Chlorination tests were performed in 40-mL brown glass ampoule bottles at a controlled room temperature (23.0 ± 0.2 °C) and under a headspace-free and light-free condition. In a typical run, an appropriate dose of chlorine was added to each model precursor solution (0.05 mM) to achieve the same molar ratio of chlorine (Cl_2_) to model precursor nitrogen atom (Cl_2_/N in model precursor = 20) at the beginning of the chlorination reaction. Solution pH was maintained in buffer solution (10 mM), which were prepared from phosphate and carbonate salts. If needed, NaOH and HCl were used to adjust the pH to a desirable level. To examine the speciation of HAcAms, an appropriate dose of bromide (potassium bromide) was added to each model precursor solution (0.05 mM), to achieve the same molar ratio of bromide to model precursor nitrogen atom (bromide/N in model precursor = 2) at the beginning of the chlorination reaction. The Cl_2_/N in model precursor ratio of 20 and bromide/N in model precursor ratio of 2 were selected in order to apply more realistic process conditions^7^,^10^,^11^,^12^,^13^,^19^,^20^. To quench the chlorination reaction at designated times, the disinfectant residual was quenched with a stoichiometric amount of ascorbic acid. The quenched solution was analyzed as soon as possible after collection. Detailed information on the experimental procedure is available elsewhere^15^.

### Analysis

In the analysis of 9 HAcAms, a simultaneous determination method for HAcAms, combining solid-phase extraction (SPE) enrichment, high-performance liquid chromatography (HPLC) separation, and triple quadrupole MS (tqMS) with atmospheric pressure chemical ionization (APCI), using selective reaction monitoring (SRM) in the positive mode, was developed.

The SPE performance of neutral (HLB), cation-exchanging (MCX, WCX), and anion-exchanging (MAX, WAX) OASIS polymers supplied by Waters (Milford, MA, USA) has been studied recently^8^. The neutral solutes HLB had the highest SPE performance (highest recoveries) for the nine HAcAms and was selected as the SPE sorbent for this method.

After SPE enrichment, an HPLC (e2695) from Waters (Milford, MA), employing a Hypersil GOLD C18 packed column (100 × 2.1 mm i.d., 5 μm) with a Hypersil GOLD precolumn (10 × 2.1 mm i.d., 5 μm) (Thermo Scientific; Waltham, MA) was used for separation. The 9 HAcAms were separated by LC in 9.0 min.

After the HPLC separation, a tqMS (TSQ Quantum Access MAX) from Thermo Scientific (Waltham, MA) was used to detect the 9 HAcAms by positive APCI combined with the SRM mode. The optimal operating parameters were as follows: discharge current at 4.0 μA, vaporizer temperature at 350 °C, sheath gas pressure at 40 psi, capillary temperature at 250 °C, and collision pressure at 1.5 m Torr. Transition ions, collision energy, and tube lens offset were optimized for individual analytes, as shown in Supplementary Information (SI) Table S1. The intraday and interday instrument precision were calculated by the relative standard deviations (RSDs) at three concentration levels (0.1, 1, 10 μg/L) for each HAcAm within the linear ranges. The intraday and interday RSDs (n = 5) for each HAcAm were generally lower than 10%. The details of the HAcAm and other N-DBP analyses are presented elsewhere^21^ and are summarized in the SI. The HAcAm yield was the molar ratio of the formed HAcAm to the initial concentration of selected free or combined AAs (equation 1). At the Cl_2_/N in model precursor (molar ratio) of 20, the AAs could be consumed completely at a short period (<60 min)^17^,^18^,^22^, thereby the initial AA molar concentration can be regarded as the consumed AA molar concentration.


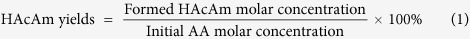


## Results and Discussion

### Impacts of peptide bonds in dipeptides on the HAcAm formation during chlorination

**Figure 2:**
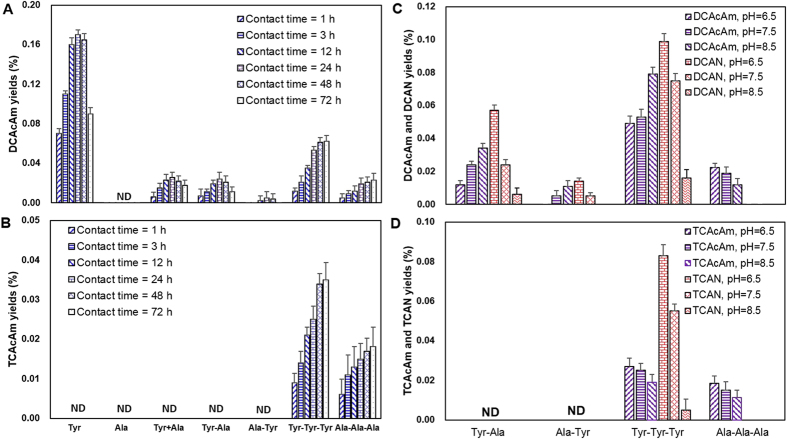
Formation of HAcAms during chlorination of the selected AAs at different contact times (DCAcAm [A] and TCAcAm [B]) and pH levels (DCAcAm and DCAN [C] and TCAcAm and TCAN [D]). AAs molar concentration = 0.05mM, Cl_2_/N in model precursor (molar ratio) = 20, pH=7.5, except as noted. ‘Tyr+Ala’ represent the mixed solution of fee Tyr and free Ala ([Tyr] = [Ala] = 0.05 mM). The bars represent the standard deviation of replicate measurements (n = 3).

Figure 2 shows the time- and pH-dependent formation of DCAcAm and TCAcAm during chlorination of free AAs and combined AAs at a Cl_2_/N in model precursor (molar ratio) of 20. As seen from Fig. 2A, the concentrations of DCAcAm formed from free Tyr, Tyr+Ala, and two dipeptides (Tyr-Ala and Ala-Tyr) initially increased and then decreased with the contact time from 1 to 72 h, and peaked at 0.170%, 0.026%, 0.024%, and 0.005% at 24 h, respectively. The decrease of HAcAm yields after 24 h was probably because the residual chlorine accelerated the decomposition rate of HAcAms^22^. The mixed ‘Tyr+Ala’ formed less DCAcAm than free Tyr, which implied the presence of Ala (Non-HAcAm precursor)^18^ in the water solution suppressed the formation of DCAcAm from Tyr (Non-HAcAm precursor)^17^ upon chlorination, probably due to the difference of chlorine demand for Ala and Tyr.

In contrast, TCAcAm was not detected during chlorination of Tyr, Ala, Tyr-Ala, or Ala-Tyr (Fig. 2B). This results is in agreement with the previous study which found that HAcAm precursors in natural waters more easily form DCAcAm than TCAcAm^23^. Ala-Ala was similar with free Ala, which cannot form DCAcAm and TCAcAm. As shown in Fig. 2C, DCAcAm yields continuously grew with the increasing pH from 6.5 to 8.5 for Tyr-Ala and Ala-Tyr, whereas DCAN yields generally dropped with the pH increase. The formation and degradation patterns of DCAcAm may be ascribed to the hydrolysis of DCAN and DCAcAm. DCAN is relatively stable at pH 6.5, but can hydrolyze to form DCAcAm as the alkalinity increases (Equation S1)^24^. Even though DCAcAm can hydrolyze to form DCAA, the hydrolysis rate of DCAcAm was generally less than the formation rate of DCAcAm from DCAN hydrolysis^15^,^21^.

**Figure 3:**
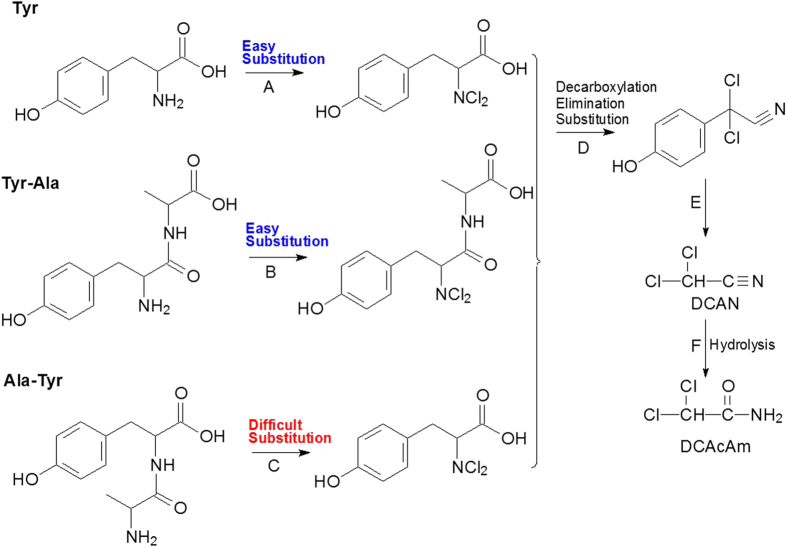
Proposed formation pathway of HAcAms from free Tyr, Tyr-Ala, and Ala-Tyr.

Previous studies have found that free Tyr may form DCAcAm through initial substitution (reaction A in Fig. 3), decarboxylation, elimination and further substitution reactions (reaction D in Fig. 3), as well as hydrolysis reaction (reaction F in Fig. 3)^17^. In this study, free Tyr in the mixed ‘Tyr+Ala’ solution yielded DCAcAm at similar concentrations as Tyr-Ala, which formed more DCAcAm than Ala-Tyr, probably because the protection of the amino group in Ala-Tyr inhibited the formation of organic chloramines by initial substitution (reaction C in Fig. 3) as the first step for the formation of N-DBPs^25^,^26^.

### Impacts of peptide bonds in tripeptides on the HAcAm formation during chlorination

Figure 2 also presents the formation of DCAcAm and TCAcAm from the chlorination of two tripeptides (Tyr-Tyr-Tyr and Ala-Ala-Ala). Of note, Ala-Ala-Ala substantially transformed to DCAcAm, whereas it is known that free Ala and Ala-Ala cannot form DCAcAm above the detection limit during chlorination in the study, which was also found in the previous study^15^. Moreover, unlike free AAs (Tyr and Ala) and dipeptides (Ala-Ala, Tyr-Ala and Ala-Tyr), Tyr-Tyr-Tyr and Ala-Ala-Ala both produced TCAcAm. The concentrations of DCAcAm and TCAcAm from the chlorination of Tyr-Tyr-Tyr and Ala-Ala-Ala increased over the entire study time from 1 to 72 h (Fig. 2A,B).

**Figure 4:**
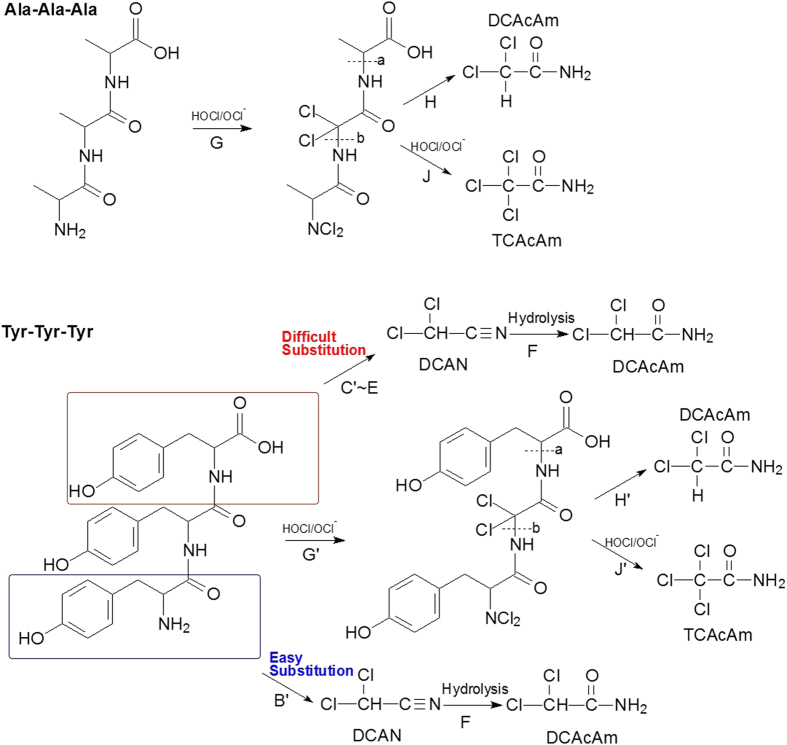
Proposed formation pathway of HAcAms from free Ala-Ala-Ala and Tyr-Tyr-Tyr.

As shown in Fig. 2C,D, Ala-Ala-Ala could not form DCAN and TCAN above the detection limit at three selected pH levels, and, DCAcAm and TCAcAm both decreased as pH increased. This finding indicated that the formation of DCAcAm was independent of DCAN hydrolysis, which was different from the previous HAcAm formation pathway (Fig. 3). Previous studies had found that chlorine substitution reaction would take place on the nitrogen atom at the amino-terminal function^27^. However, no chlorine reactivity with the nitrogen atom at the peptide bond or the carboxyl-terminal residue was previously shown^28^,^29^,^30^. Also, it has been appreciated that the hydrogen atoms of the methyl group between two carbonyl function groups are readily dissociated and the chlorine substitution is thus rapid^31^,^32^. Accordingly, the methyl group between the two carbonyl function groups in Ala-Ala-Ala and Tyr-Tyr-Tyr could be substituted by chlorine (Reactions G and G’ in Fig. 4), and probably form a small quantity of DCAcAm and TCAcAm through C–N bond breaking (bonds a and b)^33^,^34^,^35^ and further chlorine substitution and (Reactions H and J, H’ and J’ in Fig. 4). It should be noted that the proposed formation pathway of HAcAms during chlorination of oligopeptides was a speculative side reaction pathway. More research is needed to confirm the hypothesis.

Under a typical water treatment relevant pH, Cl_2_ completely hydrolyzes, and the primary active chlorine species include HOCl and OCl^−^ (Equation S2). Since the equilibrium constant (K) for Equation (S3) is 2.9 × 10^−8^ at 25 °C, HOCl and OCl^−^ are the dominant species at pH 4.0–8.0 and 8.0–10.0, respectively^32^. Since HOCl is more reactive than OCl^−^ in water^32^, the chlorine substitution was faster at lower pH level (pH = 6.5) than at higher pH level (pH = 8.5). Consequently, this probably resulted in more production of DCAcAm and TCAcAm from Ala-Ala-Ala at pH 6.5 than at pH 8.5. Similarly, more TCAcAm was formed from chlorination of Tyr-Tyr-Tyr at lower pH level. However, DCAcAm formation from Tyr-Tyr-Tyr did not show a similar pattern with TCAcAm, probably because the formation of DCAcAm was not only from the chlorine substitution reaction adjacent to peptide bond (Reaction G’ in Fig. 4), but also from the hydrolysis of DCAN (Reaction B’ in Fig. 4) which is similar to the formation of DCAcAm from Tyr-Ala (Reaction B in Fig. 3).

### Impacts of peptide bonds on HAcAm speciation during chlorination

**Figure 5:**
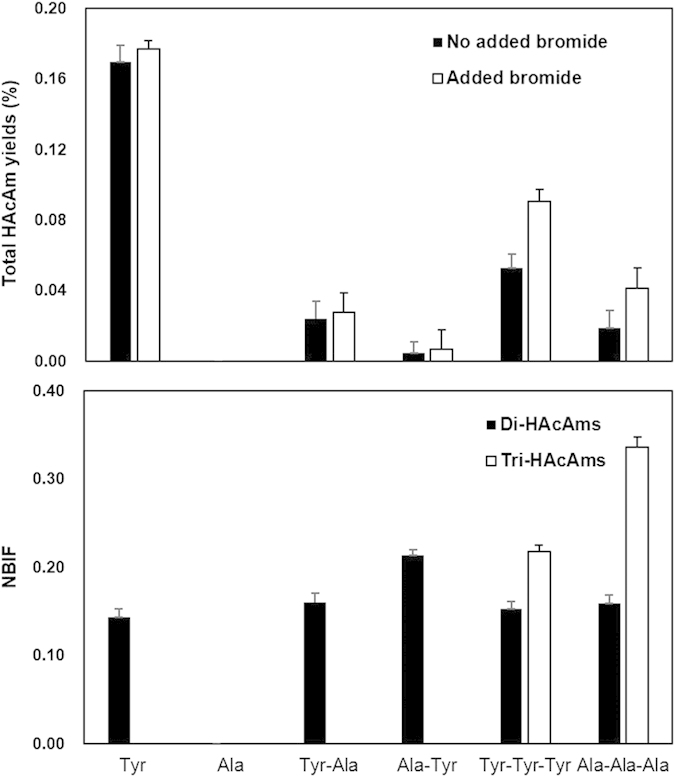
Total yields (A) and NBIF values (B) of HAcAms during chlorination of the selected AAs. AAs molar concentration = 0.05mM, Cl_2_/N in model precursor (molar ratio) = 20, pH = 7.5, bromide/N in model precursor (molar ratio) = 2, except as noted. The bars represent the standard deviation of replicate measurements (n = 3).

The formation of brominated HAcAms is of particular interest, since they are more toxic than their chlorinated analogues^2^,^6^. In order to examine the effect of peptide bonds on HAcAm speciation from the selected free AAs and combined AAs (oligopeptides), the AA water solution was added with bromide. As shown in Fig. 5A, bromide did not significantly change the yields of total HAcAms from chlorination of free Tyr and dipeptides (Tyr-Ala and Ala-Tyr), but it increased the yields of total HAcAms from the tripeptides (Tyr-Tyr-Tyr and Ala-Ala-Ala). Generally, bromide can form HOBr during chlorination as shown in Equation (S4)^36^,^37^. Compared with HOCl, HOBr has a low dissociation of degree and a high oxidizability than HOCl^20^,^32^, and thus the methyl group between the two carbonyl functions in Ala-Ala-Ala and Tyr-Tyr-Tyr was more easily substituted by HOBr than HOCl (Reactions G and G’ in Fig. 4), and probably form more brominated HAcAms.

In order to further investigate the speciation of HAcAms from the selected free AAs and oligopeptides, bromine incorporation factors (BIF) for HAcAms were calculated as in studies of other DBPs^38^,^39^, the BIF being used as an index to describe the proportion of the HAcAms that can be partially or totally substituted with bromine atoms. The following formulae were applied to calculate BIF (equations (2) and (3)), where all concentrations are on a molar basis:


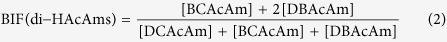



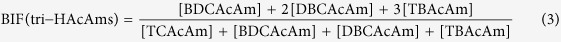


BIFs for di-HAcAms ranged from 0 (all DCAcAm) to 2 (all DBAcAm), and BIFs for tri-HAcAms ranged from 0 (all TCAcAm) to 3 (all TBAcAm). A tri-HAcAm BIF of 1.0 means that the average tri- HAcAm species is BDCAcAm. To better compare BIFs, each was normalized by the number of halogens, where the normalized BIF (NBIF) for di-HAcAms was its BIF divided by 2 and the NBIF for tri-HAcAms was its BIF divided by 3 (i.e., both NBIFs range from 0 to1), as shown in Fig. 5B. The NBIF values for all selected AAs were all between 0.1 and 0.35, which is in agreement with a recent study^23^. The recent study investigated the NBIFs of HAcAms formed from the chlorination of several natural waters containing bromide at 50~200 μg/L^23^. Of note, there was more bromine incorporation into tri-HAcAms than in di-HAcAms during chlorination of Tyr-Tyr-Tyr and Ala-Ala-Ala. For chlorinated HAcAms, more di- HAcAms (0.043% for Tyr-Tyr-Tyr, 0.015% for Ala-Ala-Ala) were formed than tri-HAcAms (0.018% for Tyr-Tyr-Tyr, 0.011% for Ala-Ala-Ala). Whereas the yields of brominated tri-HAcAms (0.029% for Tyr-Tyr-Tyr, 0.019% for Ala-Ala-Ala) were higher than that of brominated di-HAcAms (0.019% for Tyr-Tyr-Tyr, 0.007% for Ala-Ala-Ala). Especially for Ala-Ala-Ala, the NBIF (tri-HAcAms) was significantly higher than NBIF (di-HAcAms). As discussed earlier, unlike Tyr-Tyr-Tyr, Ala-Ala-Ala formed di-HAcAms and tri-HAcAms only through a single halogenating reaction adjacent to the peptide bond (Fig. 4), and the yields of di-HAcAms (DCAcAm) and tri-HAcAms (TCAcAm) were similar (about 0.02%) when bromide was not added (Fig. 2A,B). This results indicated that brominated tri-HAcAms was more easily to be formed by halogen (chlorine and bromine) substitution reaction adjacent to the peptide bond than brominated di-HAcAms. As reported, brominated tri-HAcAms are more cytotoxic and genotoxic than their di-HAcAm analogues, therefore, DWTPs should give attention to the formation of HAcAms in those algal- and wastewater-impacted waters rich in peptide bonds and bromide.

## Conclusions

The use of wastewater-impacted water as drinking water sources increases concerns of the exposure of N-DBPs (e.g., HAcAms), because wastewater-induced DON plays a key role as N-DBP precursors. In the previous studies to investigate the nitrogen origin of HAcAms, α-amine terminus of free AAs was focused on. However, free AAs only accounts for a small fraction of dissolved organic nitrogen (DON) pool in source waters. Due to the low HAcAm yields (<0.2%) from the chlorination of AAs, low-level free AAs cannot supply enough nitrogen in HAcAms in chlorinated drinking water. Combined AAs could be an important nitrogen source in HAcAm formation during chlorination, especially in algal- and wastewater-impacted water.

This study firstly revealed that the HAcAm formation from AAs in more complex structures (oligopeptides) was different to the formation from free AAs. Compared to free AAs, the peptide bonds in oligopeptides, including dipeptides and tripeptides, reduced the contribution of the combined AAs on the DCAcAm formation. However, the peptide bond in tripeptides produced more TCAcAm compared to free AAs which were unable to form TCAcAm. These results implied that the peptide bonds contributed to the formation of HAcAms, and thus played a likely more important role in prediction of specific N-DBPs (e.g., HAcAm) concentrations upon chlorination.

Apart from the most frequently and abundantly detected di-HAcAms, DWTPs should also consider the formation of tri-HAcAms in those algal- and wastewater-impacted waters rich in peptide bonds and bromide, because bromide probably promoted the formation of total HAcAms (esp., brominated tri-HAcAms), and bromine-containing tri-HAcAms have been shown to be more cytotoxic and genotoxic than di-HAcAm analogues. A benefit of improving the removal of combined AAs before chlorination disinfection is reduced formation of HAcAms and thus reduced health concerns.

## Additional Information

**How to cite this article**: Chu, W. *et al.* Peptide bonds affect the formation of haloacetamides, an emerging class of N-DBPs in drinking water: free amino acids versus oligopeptides. *Sci. Rep.* **5**, 14412; doi: 10.1038/srep14412 (2015).

## Supplementary Material

Supplementary Information

## Figures and Tables

**Figure 1:**
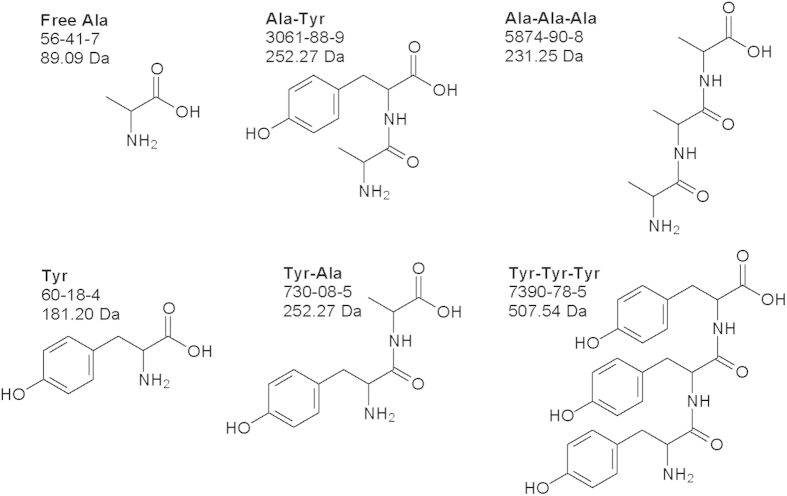
Chemical structures of selected free and combined AAs in the study.
