# Supplementary material for: Peptide bonds affect the formation of haloacetamides, an emerging class of N-DBPs in drinking water: free amino acids versus oligopeptides
Source: Sci Rep. 2015 Sep 23;5:14412. doi: 10.1038/srep14412 (PMC4585778; doi:10.1038/srep14412)
Supplement: Supplementary Information [file srep14412-s1.doc]

# **Supplementary Information**

**Peptide bonds affect the formation of haloacetamides, an emerging class of N-DBPs in drinking water: free amino acids versus oligopeptides**

Wenhai Chu^1^, Xin Li^1^, Naiyun Gao^1^, Yang Deng^2^, Daqiang Yin^1^, Dongmei Li^1^, Tengfei Chu^1^

^1^*State Key Laboratory of Pollution Control and Resources Reuse, College of Environmental Science and Engineering, Tongji University, Shanghai, 200092, China*

^2^ *Department of Earth and Environmental Studies, Montclair State University, Montclair, NJ 07043, USA*

Correspondence and requests for materials should be addressed to W.H.C. (feedwater@126.com)

Analytical methods

Chemical reaction equations

**Table S1** Transition ions of 9 HAcAms, optimized collision energy (CE), tube lens offset (TLO), and retention time (RT)

**Analytical methods**

A simultaneous determination method, combining solid-phase extraction (SPE) enrichment, high performance liquid chromatography (HPLC), and triple quadrupole mass spectrometry (tqMS) with atmospheric pressure chemical ionization (APCI), using selective reaction monitoring (SRM) in the positive mode, was used. Oasis HLB was selected as the polymeric SPE sorbent for analysis of HAcAm samples. A GAST DOA-P504-BN oil-less vacuum pump (Michigan, USA) was used to draw the water samples through the SPE column. A typical SPE run involved conditioning and equilibrating the sorbents with 10 ml of methanol, then with 10 ml of ultrapure water at a flow rate of 5 mL/min. Once methanol was added, the SPE bed was not allowed to dry before extraction of the samples. The water samples (usually 500 mL) were filtered and adjusted to pH 5±0.5 to prevent the hydrolysis of HAcAms and passed through the SPE cartridge at a flow rate of 3-5 mL/min (1 drop/s). After the extraction, the SPE column was washed with 5 mL of 95% ultrapure water/5% methanol and immediately eluted with 0.5 mL of water and 5 mL of methanol. The organic eluent was collected and concentrated down to 0.5 mL at 40°C by a pressured nitrogen gas blowing concentrator (Youcheng Union Technology Co., Ltd., UGC-12MF, Beijing, China). The extracts were then analyzed immediately by HPLC/tqMS.

An HPLC (e2695) from Waters (Milford, MA, USA) connected to a tqMS (TSQ Quantum Access MAX) from Thermo Scientific (Waltham, MA, USA) was used to determine the 13 HAcAms (in this study, nine chlorinated HAcAms were determined). Analyst software Xcalibur was used for data acquisition and analysis. A Hypersil GOLD C18 packed column (100 × 2.1 mm i.d., 5 μm) with a Hypersil GOLD precolumn (10 × 2.1 mm i.d., 5 μm) (Thermo Scientific; Waltham, MA) was used for separation. The column temperature was controlled at 40^o^C by an Alliance column heater from Waters (Milford, MA, USA). The mobile phase was composed of solvent A (ultrapure water) and solvent B (100% methanol). The solvent gradient program consisted of 5% of solvent B for 2 min, increasing solvent B from 5% to 90% over 8 min, and returning back to 5% of solvent B over 0.1 min, followed by a 5-min re-equilibration prior to the next sample injection. The flow rate was 300 μL/min. Nine HAcAms were separated by LC in 9.0 min, and the sample injection volume was 10 μL.

After the LC separation, detection was performed by positive APCI combined with the SRM mode. The optimization of MS conditions was performed by infusing a mixture of 1 mg/L of HAcAms (5% MeOH: 95% water) using a syringe pump. The optimal operating parameters were as follows: discharge current at 4.0 μA, vaporizer temperature at 350^o^C, sheath gas pressure at 40 psi, capillary temperature at 250^o^C, and collision pressure at 1.5 mTorr. Transition ions, collision energy and tube lens offset were optimized for individual analytes, as shown in SI Table S1.

Detection limits of CAcAm, DCAcAm, TCAcAm, BAcAm, DBAcAm, BCAcAm, BDCAcAm, DBCAcAm, and TBAcAm were 20, 40, 50, 20, 20, 40, 60, 30, and 30 ng/L, respectively (relative standard deviations [RSDs] were 1.0-10%).

**Chemical reaction equations**


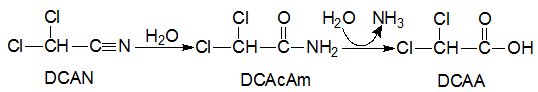
 (Equation S1)

(Equation S2)

(Equation S3)

(Equation S4)

Table S1 Transition ions of 9 HAcAms, optimized collision energy (CE), tube lens offset (TLO), and retention time (RT)

| HAcAms | Parent ion | Product ion | CE | TLO | RT (min) |
| --- | --- | --- | --- | --- | --- |
| CAcAm | 94.1 | 58.2 | 14 | 75 | 1.50 |
|  |  | 59.3 | 20 |  |  |
| DCAcAm | 128.1 | 64.2 | 17 | 80 | 2.25 |
| TCAcAm | 162.0 | 62.4 | 39 | 60 | 8.22 |
|  |  | 83.2 | 18 |  |  |
|  |  | 98.2 | 15 |  |  |
| BAcAm | 138.0 | 43.2 | 29 | 84 | 1.56 |
|  |  | 58.9 | 15 |  |  |
|  |  | 121 | 19 |  |  |
| DBAcAm | 217.9 | 59.2 | 21 | 46 | 1.73 |
|  |  | 186 | 6 |  |  |
| BCAcAm | 174.0 | 76.1 | 37 | 81 | 2.51 |
|  |  | 93.2 | 16 |  |  |
|  |  | 95.4 | 18 |  |  |
| BDCAcAm | 205.9 | 82.1 | 34 | 68 | 8.83 |
|  |  | 98.1 | 19 |  |  |
|  |  | 110 | 26 |  |  |
|  |  | 127 | 14 |  |  |
| DBCAcAm | 251.9 | 76.1 | 37 | 58 | 3.07 |
|  |  | 93.1 | 20 |  |  |
|  |  | 220 | 6 |  |  |
| TBAcAm | 297.8 | 139 | 19 | 81 | 3.48 |
|  |  | 200 | 15 |  |  |
|  |  | 217 | 11 |  |  |
|  |  | 266 | 6 |  |  |
